# Cadmium and Breast Cancer: Exposure Associated with Basal-Like Phenotype

**DOI:** 10.1289/ehp.117-a552b

**Published:** 2009-12

**Authors:** Julia R. Barrett

**Affiliations:** **Julia R. Barrett**, MS, ELS, a Madison, Wisconsin–based science writer and editor, has written for *EHP* since 1996. She is a member of the National Association of Science Writers and the Board of Editors in the Life Sciences

Cadmium has been linked with several human diseases including chronic kidney disease and cancer. As a carcinogen, cadmium targets several sites that are considered endocrine-sensitive, and some data suggest the breast may be among them. Although cadmium has been hypothesized to act as a metalloestrogen—a metal that triggers an estrogen-like reaction—research to date has not confirmed this as a mechanism linking cadmium and breast cancer. Additionally, although many breast cancers are estrogen-dependent, some of the most difficult-to-treat cases are not. A new study finds that cadmium can induce malignant transformation in breast cells *in vitro* regardless of the absence of estrogen receptors, strengthening evidence that cadmium exposure may be a factor in breast cancer, a leading cause of cancer deaths among women **[*EHP*** **117:1847–1852; Benbrahim-Tallaa et al.]**.

MCF-10A cells, which are derived from normal human breast epithelium, were grown with either no cadmium exposure or continuous cadmium exposure (2.5 μM) for up to 40 weeks. Positive controls included MCF-7 human breast cancer cells (which express the estrogen receptors ER-α and ER-β) and SKBR3 breast cancer cells (which express HER2, a receptor that can be overexpressed in certain malignant breast cancer cells). In contrast, MCF-10A cells do not express ER-α, ER-β, or HER2 proteins, although expression can be acquired in carcinogenesis.

Chronic cadmium exposure of the MCF-10A cells yielded increased expression of matrix metalloproteinase-9, an enzyme that facilitates tumor cell invasion. These cells also formed cell mounds, indicating a loss of contact inhibition (the natural process of cell growth stopping once a certain density of cells is reached). When these transformed cells were implanted in mice, they formed highly aggressive tumors that demonstrated metastatic potential.

Transformed MCF-10A cells remained negative for ER-α and ER-β and also lacked HER2 protein. However, metallothionein, typically overexpressed in ER-negative breast cancers, was elevated as were several other breast cancer markers. These characteristics collectively suggest that cadmium could be a risk factor for a basal-like breast cancer phenotype, which is clinically associated with a higher risk of relapse after treatment and lower survival rates.

The precise mechanism by which cadmium may transform breast cells is unknown, but the results of this study suggest it is unlikely to be a metalloestrogenic effect via estrogen receptors. Although additional research is needed to define the mechanism, the current study provides strong evidence that cadmium may play a role in human breast cancer.

